# Regression of Cardiac Rhabdomyomas in a Neonate after Everolimus Treatment

**DOI:** 10.1155/2016/8712962

**Published:** 2016-06-27

**Authors:** Helen Bornaun, Kazım Öztarhan, Tugba Erener-Ercan, Reyhan Dedeoğlu, Deniz Tugcu, Çiğdem Aydoğmuş, Merih Cetinkaya, Sultan Kavuncuoglu

**Affiliations:** ^1^Department of Pediatric Cardiology, Kanuni Suleyman Teaching and Research Hospital, 34303 Istanbul, Turkey; ^2^Department of Neonatology, Kanuni Suleyman Teaching and Research Hospital, 34303 Istanbul, Turkey; ^3^Department of Pediatric Cardiology, Cerrahpasa Medical Faculty, Istanbul University, 34098 Istanbul, Turkey; ^4^Department of Pediatric Hematology-Oncology, Kanuni Suleyman Teaching and Research Hospital, 34303 Istanbul, Turkey

## Abstract

Cardiac rhabdomyoma often shows spontaneous regression and usually requires only close follow-up. However, patients with symptomatic inoperable rhabdomyomas may be candidates for everolimus treatment. Our patient had multiple inoperable cardiac rhabdomyomas causing serious left ventricle outflow-tract obstruction that showed a dramatic reduction in the size after everolimus therapy, a mammalian target of rapamycin (mTOR) inhibitor. After discontinuation of therapy, an increase in the diameter of masses occurred and everolimus was restarted. After 6 months of treatment, rhabdomyomas decreased in size and therapy was stopped. In conclusion, everolimus could be a possible novel therapy for neonates with clinically significant rhabdomyomas.

## 1. Introduction

Primary cardiac tumors are rare during childhood. Cardiac rhabdomyoma is the most common benign pediatric tumor of the heart and accounts for approximately 10% of the tumors seen in the fetus and neonate [1–3]. It frequently occurs in association with tuberous sclerosis (TSC), an autosomal dominant disorder that results in abnormal cellular proliferation and differentiation which are responsible for hamartomatous lesions that can affect the brain, kidney, heart, and lungs [1–4]. The majority of patients with TSC have inactivating mutations in either TSC1 (9q34.3) or TSC2 (16p13.3) gene, resulting in constitutive activation of mTOR, a protein which is, when activated, responsible for a proliferative cellular response and cell activation [5]. It has been appreciated that mTOR inhibitors are antiproliferative agents. Everolimus is a derivative of sirolimus, which is approved by the FDA for the treatment of patients with subependymal giant-cell astrocytomas (SEGAs) associated with TSC, and acts similarly to sirolimus as an inhibitor of mTOR; hence, an everolimus trial is currently proceeding to attempt to treat symptomatic patients with inoperable CRs [5, 6]. The hemodynamic impact of CRs depends on the location and size of the mass and the presence of arrhythmias, systolic and diastolic dysfunction, and ventricular mechanical obstruction which can be very problematic even leading to death [1–5]; thus, a good clinical evaluation is critical for the decision of surgery and/or follow-up in the neonate with CRs [3]. Herein, we report a newborn with inoperable multifocal CRs and significant regression of cardiac masses after receiving everolimus.

## 2. Case Presentation

**Figure 1:**
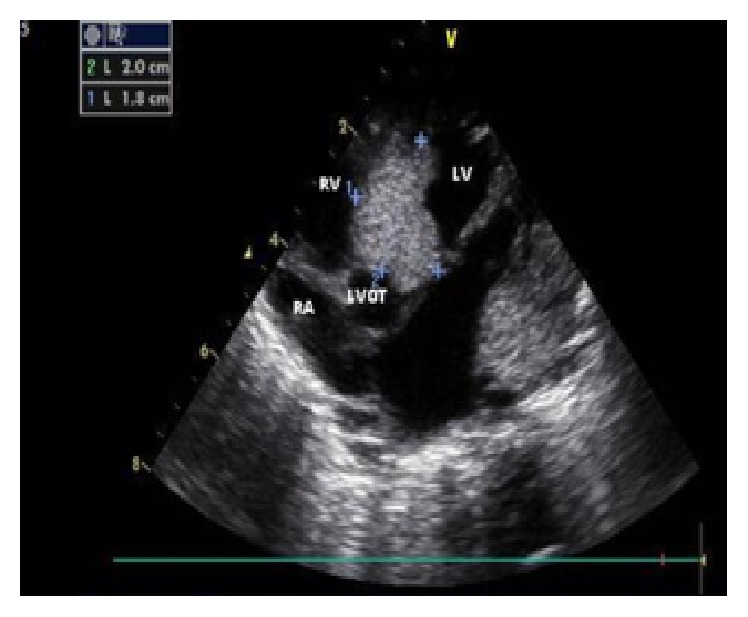
Echocardiography showing a great mass in the LVOT region of the heart (postnatal in the first day).

The female infant was delivered by cesarean section at 38 weeks of gestation as the first child of nonconsanguineous healthy parents. She was admitted to the neonatal intensive care unit due to respiratory distress for which nasal CPAP was applied for one day. The fetal ultrasonography of the patient at 26 weeks revealed multiple CRs in both ventricles. The prenatal cranial MRI revealed multiple hamartomas (6 to 15 mm in diameter) in the subependymal area. Birth weight, length, and head circumference were 3500 g (50 percentile), 50 cm (50 percentile), and 34.5 cm (50 percentile), respectively. In cardiovascular examination, a 3/6 systolic ejection murmur was heard over the mesocardiac area. The postnatal echocardiography (ECHO) revealed multiple rhabdomyomas in both ventricles with evidence of left ventricle outflow-tract (LVOT) continuous Doppler (CW Doppler) measured 66 mmHg gradient between left ventricle and aorta. The mass located in the LVOT was very large (diameter: 1.3 cm^2^) and had extensive intramural components (Figure 1). There were also smaller masses in different regions of the right ventricle (RV) that caused mild stenosis in the right ventricle outflow tract (RVOT). Postnatal cranial MRI revealed multiple calcified hamartomas along the periventricular zone.

On the seventh day of life, she developed poor sucking, cyanosis, and hypotonia. Oxygen saturation levels started to decrease. Hematologic profile, acute phase reactants (C-reactive protein, sedimentation rate), and ammonia levels were normal. Control ECHO revealed a significant increase in the size of the rhabdomyomas and an increase in the pressure of the LVOT obstruction CW Doppler gradient (max: 80 mmHg). The electrocardiography (ECG) was abnormal showing marked ST depression and voltage criteria for left ventricle hypertrophy (LVH).

**Figure 2:**
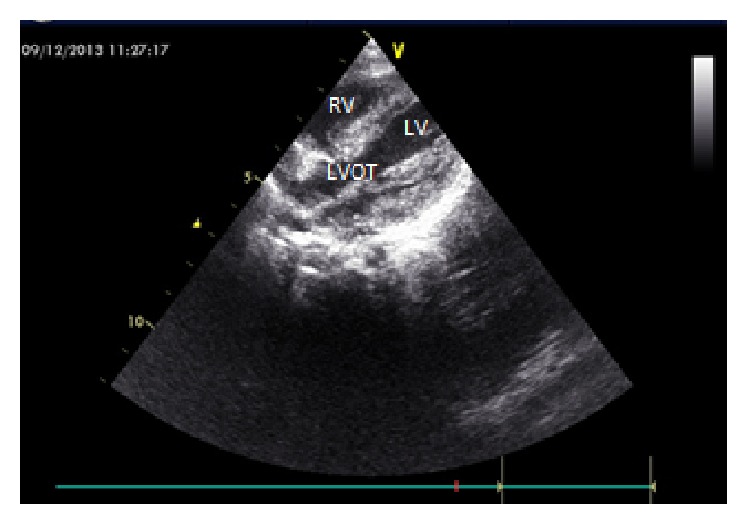
Echocardiography showing a significant diminish in the mass size (in the postnatal 28 days).

Dopamine and prostaglandin E1 (0.01 mcg/kg/min) infusions were begun to maintain systemic blood flow. Everolimus therapy at a dose of 2 × 0.25 mg twice a week was started since the patient became symptomatic and the intracardiac mass was not amenable to surgery. During the fourth week of the everolimus treatment, a significant reduction in the size of the rhabdomyomas was observed (Figure 2). After one-month everolimus treatment, due to elevated serum triglyceride levels (TG: 560 mg/dL), therapy was stopped as the diameter of the mass in the LVOT reduced with resolution of LVOT obstruction. In follow-up, the physical examination revealed an increase in murmur intensity, and significant regrowth of all cardiac masses was observed by ECHO 10 days after cessation of therapy. Everolimus treatment was restarted at the same dose. During the second course of therapy, serum TG and total cholesterol levels increased (384 mg/dL and 184 mg/dL, resp.). However, after starting omega-3 treatment orally, the TG levels returned to normal.

Molecular genetic studies revealed a mutation in TSC1 in the infant, but parents' genetic analysis was normal. An ophthalmologic inspection revealed pigmented retinal lesions in both eyes. A few hypopigmented skin lesions appeared on the trunk and extremities.

The infant's serum everolimus levels in the course of therapy ranged between 0.4 and 2.6 ng/mL (normal: 2.6–6.1 ng/mL). Control ECHO revealed a decrease in the diameter of cardiac masses and increase in interventricular septum (IVS) thickness. After 6 months of everolimus therapy, a repeat ECHO showed a marked decrease in the size of cardiac masses with a significant thickening in the IVS diameter (from 6 mm to 12 mm) and left ventricular posterior wall. Everolimus therapy was stopped by tapering the dose in a 1.5-month period to prevent a rebound increase in mass size and to prevent late side effects of the drug. She was discharged from the hospital after cessation of the therapy. The patient is currently 1 year old and is under close follow-up. There has been no significant change in the diameters of the CRs after cessation of therapy.

## 3. Discussion

Congenital CRs comprise 45–75% of the cardiac tumors [7]. Most of the cases are asymptomatic. Patients with symptomatic, inoperable CRs may be candidates for everolimus treatment. Treatment of rhabdomyomas with everolimus is a rare phenomenon, with only 17 cases reported in the literature [1–9].

One of the most important features of CRs is spontaneous regression. Transplacentally transmitted maternal estrogens are reported to be responsible for the growth of CRs in utero. As the effects of maternal estrogens diminish, rhabdomyomas regress. However, cardiac rhabdomyomas may cause serious clinical problems before regression such as severe arrhythmias, ventricular inflow and outflow-tract obstructions, and congestive heart failure. These patients may require medical care for the treatment of arrhythmias and heart failure and surgical repair for the correction of severe mechanical obstructions. Our patient had a very large mass that had infiltrated most of the LVOT region intramurally and caused severe obstruction, so it was not suitable for surgical intervention. Therefore, everolimus treatment was started on the seventh day of life. Shortly after treatment, symptomatic CRs demonstrated a dramatic reduction in size and effective systemic blood flow was resumed with an increase in O_2_ saturation levels so that no further PGE1 infusion was needed. However, shortly after stopping everolimus treatment, masses began to enlarge. Studies have reported that mTOR inhibitors were successful in the treatment of patients with TSC noting that CRs did not relapse after cessation of therapy. A study conducted by Tiberio et al. [4] in 2011 reported complete resolution of a large left ventricular mass in a seven-year-old patient with TSC treated by everolimus for subependymal giant-cell astrocytomas. In their study, blood levels of everolimus ranged between 2.3 and 7.1 ng/mL (therapeutic dose, 5–15 ng/mL). In our study, blood levels ranged between 2.2 and 2.6 ng/mL which were below therapeutic levels. Since the patient was clinically stable, we did not attempt to increase the dose of the drug to attain therapeutic blood levels in consideration of the possible side effects of the drug. Tiberio et al. [4] and Demir et al. [6] also did not report recurrence of the masses after the termination of the therapy. In our study, we observed rapid response to the treatment. However, shortly after the cessation of therapy, cardiac masses began to expand in size. We hypothesized that if we had continued the therapy for a longer period, or if the cessation of therapy had been done by slow decrements in dose, there might had not been any need for a second course of therapy, and the masses would had not recurred. There was a change in the lipid profile during the first month of the therapy. In accordance with the results of the study conducted by Demir et al. [6], TG and cholesterol levels increased. Another significant side effect was observed in lymphocyte subgroups, similarly with the results of the study by Demir et al. [6]. These findings were managed by everolimus dose adjustment.

Another significant finding observed during therapy was the thickening of interventricular septum and left ventricular posterior wall. Serial echocardiograms revealed thickening of the heart walls and an increase in IVS from 6 mm to 11 mm. Significant cardiac hypertrophy observed in our case was believed to be a side effect of the drug. So far, FDA has reported only one case of cardiac hypertrophy related to everolimus use. In our case, heart wall thickening normalized only after drug discontinuation. Further studies are needed in order to investigate the efficacy and adverse reactions of everolimus for the treatment of cardiac rhabdomyomas.

In conclusion, we presented a novel therapeutic option for the treatment of symptomatic rhabdomyomas in the newborn period that were not amenable to surgery. However, further studies are needed to demonstrate the efficacy and safety of the use of everolimus in the treatment of CRs.
